# Growth in Children with Cerebral Palsy during five years after Selective Dorsal Rhizotomy: a practice-based study

**DOI:** 10.1186/1471-2377-10-57

**Published:** 2010-07-01

**Authors:** Lena Westbom, Annika Lundkvist Josenby, Philippe Wagner, Eva Nordmark

**Affiliations:** 1 Division of Paediatrics, Department of Clinical Sciences (Lund), Lund University, Lund, Sweden; 2 Division of Physiotherapy, Department of Health Sciences, Lund University, Lund, Sweden; 3 Swedish National Competence Centre for Musculoskeletal Disorders (NKO), Department of Orthopaedics, Skåne University Hospital, SE 221 85 Lund, Sweden; 4 Children's Hospital, Skåne University Hospital, SE 221 85 Lund, Sweden

## Abstract

**Background:**

Overweight is reported as a side effect of SDR. The aims were to study the development of weight, height and body mass index (BMI) during five years after SDR.

**Methods:**

This prospective, longitudinal and practice-based study included all 56 children with CP spastic diplegia undergoing SDR from the start in March 1993 to April 2003 in our hospital. The preoperative Gross Motor Function Classification System (GMFCS) levels were I-II in 17, III in 15, IV-V in 24 children. Median age at SDR was 4.3 years (range 2.4-7.4 years). Weight and height/recumbent length were measured. Swedish growth charts for typically developing children generated weight, height and BMI z-scores for age and gender.

**Results:**

The preoperative median z-scores were for height -1.92 and for body mass index (BMI) -0.22. Five years later, the median BMI z-score was increased by + 0.57 (p < 0.05). The occurrence of thinness (BMI < -2 SD) was decreased (n.s.) and obesity (BMI > + 2 SD) increased (p < 0.05). Baseline BMI and age at the start of follow-up influenced the BMI change during the five years (p < 0.001 and p < 0.05 respectively).

The individual growth was highly variable, but a tendency towards increasing stunting with age was seen in severe gross motor dysfunction (GMFCS levels IV-V) and the opposite, a slight catch-up of height in children with walking ability (GMFCS levels I-III).

**Conclusions:**

These are the first available subtype- and GMFCS-specific longitudinal growth data for children with CP spastic diplegia. Their growth potential according to these data should be regarded as a minimum, as some children were undernourished. It is unknown whether the spasticity reduction through SDR increased the weight gain velocity, or if the relative weight increase was part of the general "obesity epidemic".

For some children the weight increase was highly desirable. In others, it resulted in overweight and obesity with risk of negative health effects. Weight and height should be monitored to enable early prevention of weight aberrations also causing problems with mobility, activity and participation.

## Background

Children with cerebral palsy (CP) are generally short and many, especially non-ambulating children with CP, are severely underweight and stunted. It is unclear how much cerebral palsy per se brings leanness and short stature [1-3]. Other factors contribute. Dysphagia and other feeding problems with malnutrition are probably the most important obstacle to exploiting the intrinsic growth potential in many children and adolescents with CP [4-6].

Energy expenditure in spastic CP is not well understood. Adaptation to low food intake, altered regulation of basal metabolism, reduced lean body mass, and decreased mobility are suggested to counterbalance the energy costs of spasticity [7].

On the other hand, increased weight gain velocity after reduction of muscle tone by means of intrathechal baclofen (ITB) is reported in the literature [8-12]. Roberts and co-workers observed a similar phenomenon in children whose spasticity was reduced by selective dorsal rhizotomy (SDR) [13]. Weight centiles were crossed in an upward direction as opposed to height, which may open on to obesity in the long run [14]. The weight gains post ITB and SDR happen without any known change in the eating habits of the children. Hypothetically it is due to decreased caloric demands because of the decreased muscle tone.

Reference growth curves for children with CP have been developed [3,7,15], but cannot be used as a standard reference, since many of the included children had different degrees of chronic malnutrition. The growth development in adequately nourished and otherwise healthy children with CP remains to be established [3]. In addition, growth profiles differ between gross motor function levels [16] and are conceivable for the different CP subtypes.

SDR is a safe and effective method of reducing spasticity permanently without major negative side effects in children with CP spastic diplegia. In combination with physiotherapy SDR provides lasting functional benefits in carefully selected and systematically monitored young children. Improvements over time during a five-year follow-up period are found [17].

As the goal of treatment is to improve activities and participation, a long-term follow-up of all the children is necessary to answer crucial questions about their function as adolescents and young adults. As a part of this, growth data collected during the prospective follow-up of all SDR-operated children with spastic diplegia in Lund were used to explore their pattern of growth. The aims of this study were

• to describe the growth during five years of SDR-operated children with CP spastic diplegia at different GMFCS levels

• to compare weight, height and body mass index (BMI) before and five years after SDR

• to disclose any overweight and obesity, a suspected SDR side effect, that could compromise future function and health.

## Methods

### Design

The study is part of a structured follow-up scheme in clinical practice to develop and secure the efficacy of health care interventions; a prospective long-term study of a consecutive, complete 10-year series of SDR-operated children with CP spastic diplegia.

### Patients

All children with the CP type spastic diplegia undergoing SDR at the University Hospital in Lund from the start in March 1993 to April 2003 were included. Only children with definite cerebral palsy according to common definitions [18] were selected. The predominant neurological sign was spasticity and the lower extremities were more involved than the upper [19]. The level of gross motor function was classified pre-operatively according to the Gross Motor Function Classification System (GMFCS) [20].

During the study period 56 children with spastic diplegia were operated on with SDR. Background data are presented in Table 1. Five children with functional level GMFCS I and one child with GMFCS V are presented together with children with GMFCS levels II and IV. The three children with missing weight and/or height measurements pre-operatively are presented in Table 2. In one child, with functional level GMFCS III, the pre-operative measurements used were recorded at 1.3 years of age, and the operation was performed at 3.9 years of age. All other pre-operative measurements were performed one or a few days before the SDR operation. The follow-up visits five years after the operation were performed as scheduled within ± 2 months.

**Table 1:** Patients - preoperative characteristics

	GMFCS I-II	GMFCS III	GMFCS IV-V	TOTAL
	n = 17 (30%)	n = 15 (27%)	n = 24 (43%)	n = 56
Boys	9	11	17	37
Girls	8	4	7	19
Ratio boys:girls	1.1:1	2.75:1	3.4:1	1.95:1
Gestational age (GA):				
completed weeks				
> 26	1	0	3	4
26-27	2	0	1	3
28-31	6	10	14	30
32-36	4	4	2	10
37-42	4	1	3	8
> 42	0	0	0	0
Premature. GA not specified	0	0	1	1
*Birth weight *z-score median	-0.55 (-1.38 to 0.51;	-0.75 (-1.64 to -0.58;	-0.48 (-1.08 to 0.47;	-0.75 (-1.52 to 0.40;
(interquartile; *range*)	*-3.35 to 2.86*)	*-4.14 to 0.43*) *	*-2.73 to 5.60*)**	*-4.14 to 5.6*)
Age at SDR operation, years: Median (interquartile: *range*)	4.3 (3.9 to 5.2;	5.1 (3.9 to 5.8;	3.9 (3.3 to 4.8;	4.3 (3.4 to 5.2;
	*3.3 to 7.4*)	*3.0 to 6.6*)	*2.4 to 5.9*)	*2.4 to 7.4*)

* two missing, ** three missing

**Table 2:** The three children without complete anthropometric measurements before or 5 years after SDR operation

	Boy, GMFCS IV	Girl, GMFCS IV	Boy, GMFCS IV
Gestational age, completed weeks:	38	28	30
*Birth weight *z-score	-0.15	0.54	-0.81
Age at SDR operation, years	3.4	4.5	5.8
*Weight *z-score			
First available measure	2.31 (pre SDR)	-2.64 (1/2 year post SDR)	-1.35 (pre SDR)
18 months post SDR	1.14	-2.94	-0.77
5 years post SDR	1.59	Follow-up declined	-1.16
*Height/length *z-score			
First available measure	1.53 (1/2 year post SDR)	-3.37 (1/2 year post SDR)	0.04 (1 year post SDR)
18 months post SDR	0.71	-3.39	-0.70
5 years post SDR	-0.55	Follow-up declined	-0.74
*BMI *z-score			
First available measure	2.15 (1/2 year post SDR)	-0.40 (1/2 year post SDR)	-1.21 (1 year post SDR)
18 months post SDR	0.92	-0.23	-0.47
5 years post SDR	2.20	Follow-up declined	- 0.68

### Measurements

All children were followed for five years and were examined by the same team (two physiotherapists (PTs) and two neuropaediatricians) pre-operatively and then after 6, 12, 18 months, three and five years. On all occasions the weight and height were measured in a standardized way, after training, by the same persons (EN or ALJ), when necessary together with one of the others or the child's own PT. The same equipment was used during the first nine years; different equipment was used after the clinic moved to the new children's hospital.

A calibrated standard hospital balance was used. Small children without standing ability were weighed when held in the arms of an adult. Older children without standing ability were weighed in a sit balance. Children wore only thin underwear or the weight of diapers or light clothes was subtracted.

A standard stadiometer was used for height measurements. In children without standing ability recumbent length was measured on a gym mat, using a measuring-tape. Joint range of motion was measured on all occasions and spine deformities were recorded. As reported elsewhere, contractures and spinal deformities were rare and, if present, mild [17].

Weight and height were inscribed into computerized Swedish growth charts, in which Body Mass Index (BMI) and z-scores (standard deviations, SD) for age and gender were automatically calculated [21-23]. BMI is weight (kg)/height (m) ^2^. BMI > + 1 SD was classified as overweight, BMI > + 2 SD as obesity, < -2 SD as thinness and < -3 SD as severe thinness according to the WHO child growth standards [24].

### Statistics

Statistical analysis was done by ordinary paired t-tests and multiple linear regression using the statistical programming language R [25]. BMI five years after operation was chosen as the primary end-point and correction was made for age at operation and GMFCS level.

McNemar test was used for comparison between pre- and five years postoperative overweight, obesity and thinness.

### Ethics

According to section 31 of the Swedish Health Act clinicians are obliged to secure and develop the quality and document efficacy of care, especially when introducing new treatment methods, such as SDR in 1993 [26]. Approval from an institutional review board is not required for this type of research, but patients and all data must be and are handled according to the Helsinki Declaration. Informed consent was obtained from the participating families.

## Results

### Patients

A large proportion (43%) of the children in our study had preoperative gross motor function classified at GMFCS levels IV-V, 27% at level III and 30% at levels I-II. Twice as many boys as girls were SDR-operated (37:19). The male:female ratio was 1:1 at GMFCS I-II, but increased with increasing motor disability. The majority (48/56 children) were born pre-term. Median weight z-score at birth for gender and gestational age was -0.75 compared to the median weight z-score -1.33 at the SDR-operation in the pre-school years. The total age range at operation was 2.4 to 7.4 years, median age was 4.3 years (Table 1).

The five children operated before three years of age had their gross motor function classified as GMFCS level IV and all five children operated after their 6th birthday as levels GMFCS II-III. The three children with missing preoperative data had a similar background to the rest of the operated children (Table 2).

### Weight, height and BMI

Complete anthropometric measurements, according to the SDR follow-up scheme including the preoperative and five-year postoperative visit, were recorded on 318 (95%) out of the planned 336 occasions. All 56 children had completely recorded measurements 18 months post SDR (Tables 1 &2).

Of all 56 children six (11%) had BMI z-scores below -2 (thinness) and two (4%) higher than +2 (obesity) preoperatively. In addition one child with missing preoperative height/BMI weighed + 2.3 SD preoperatively (Table 2). As a group, children at GMFCS level III were heavier but not less stunted than the other groups, resulting in a higher median BMI z-score at GMFCS level III (Table 3).

**Table 3:** Growth in SDR-operated children with CP sp diplegia

	GMFCS I-II	GMFCS III	GMFCS IV-V	TOTAL
	n = 17	n = 15	n = 24	n = 56
*Weight *z-score median				
(interquartile; *range*)				
Preoperatively	-1.48 (-2.09 to -1.11	-0.73 (-1.46 to -0.18;	-1.63 (-1.97 to -0.70;	-1.33 (-1.97 to -0.60;
	*-2.88 to 0.01*)	*-3.84 to 0.61*)	*-3.63 to 2.31*)	*-3.84 to 2.31*)
18 months post SDR	-1.32 (-1.68 to -0.91;	-1.21 (-1.67 to -0.24;	-1.25 (-2.24 to -0.46;	-1.27 (-1.97 to -0.50;
	*-2.48 to 0.10*)	*-3.68 to 0.77*)	*-4.13 to 1.29*)	*-4.13 to 1.29*)
5 years post SDR	-0.64 (-1.85 to -0.25;	-0.92 (-1.95 to 0.19;	-1.10 (-1.73 to 0.20;	-0.92 (-1.87 to 0.13;
	*-2.45 to 1.77*)	*-3.02 to 1.87*)	*-4.32 to 1.59*)	*-4.32 to 1.87*)
**Pre-5 yrs postop, change**	**0.67 **(-0.28 to 1.65;	**- 0.06 **(-0.29 to 1.28;	**0.29 **(-0.34 to 1.30;	**0.43 **(-0.34 to 1.44;
	-*1.50 to 2.84*)	-*1.75 to 2.16*)	-*1.34 to 2.23*)	-*1.75 to 2.84*)
*Height/length *z-score median				
(interquartile; *range*)				
Preoperatively	-1.76 (-2.11 to -1.40;	-2.08 (-2.80 to -1.52;	-2.05 (-2.89 to -1.26;	-1.92 (-2.6 to -1.25;
	*-3.25 to -0.56*)	*-4.00 to 0.24*)	*-3.64 to 0.59*)	*-4.00 to 0.59*)
18 months post SDR	-1.81 (-2.35 to -1.04;	-1.90 (-2.90 to -1.38;	-1.75 (-2.63 to -1.04;	-1.82 (-2.6 to -1.10;
	*-2.97 to -0.48*)	*-3.94 to 0.21*)	*-3.98 to 0.71*)	*-3.98 to 0.71*)
5 years post SDR	-1.59 (-2.26 to -1.05;	-1.81 (-2.67 to -1.38;	-2.08 (-2.54 to -0.99;	-1.77 (-2.42 to -1.07;
	*-2.89 to -0.41*)	*-3.71 to 0.15*)	*-3.93 to -0.49*)	*-3.93 to 0.15*)
**Pre-5 yrs postop, change**	**0.19 **(-0.05 to 0.47;	**0.11 **(-0.17 to 0.41;	**-0.19 **(-0.58 to 0.26;	**0.04 **(-0.36 to 0.39;
	-*0.82 to 0.65*)	-*0.80 to 1.66*)	-*1.08 to 0.85*)	-*1.08 to 1.66*)
*BMI *z-score median				
(interquartile; *range*)				
Preoperatively	-0.57 (-0.92 to 0.02;	0.79 (-0.83 to 1.51;	-0.30 (-1.30 to 0.41;	-0.22 (-1.16 to 0.57;
	*-2.73 to 1.71*)	*-2.82 to 2.51*)	*-2.56 to 2.61*)	*-2.82 to 2.61*)
18 months post SDR	-0.19 (-0.51 to 0.50;	0.87 (-0.60 to 1.20;	-0.26 (-0.99 to 0.77;	-0.17 (-0.85 to 0.90;
	*-2.12 to 1.34*)	*-1.99 to 1.49*)	*-2.90 to 3.00*)	*-2.90 to 3.00*)
5 years post SDR	0.38 (-0.72 to 1.53;	0.54 (-0.31 to 1.55;	-0.14 (-0.64 to 1.62;	0.26 (-0.67 to 1.62;
	*-1.34 to 2.27*)	*-2.67 to 3.41*)	*-3.07 to 2.77*)	*-3.07 to 3.41*)
**Pre-5 yrs postop, change**	**0.81 **(0.16 to 1.92;	- **0.23 **(-0.39 to 1.44;	**0.66 **(-0.32 to 1.67;	**0.57 **(-0.32 to 1.79;
	-*2.43 to 3.19*)	-*1.86 to 2.08*)	-*1.93 to 3.56*)	-*2.43 to 3.56*)

Anthropometric measurements in SDR-operated children with CP sp diplegia in different GMFCS levels, presented as z-scores, median (interquartile; range), of the reference population growth curves, and median change of weight, height and BMI z-scores during five years

In the total group the preoperative median z-scores were -1.33 for weight, -1.92 for height and -0.22 for BMI. During the five-year follow-up the weight z-score median increase was 0.43, the height z-score was less changed (0.04) and the BMI score median increased by 0.57. All groups did not increase their BMI during the follow-up. The median increase in BMI z-score was highest (0.81) in children with milder gross motor functional limitations, GMFCS levels I-II. In the children with the highest BMI, GMFCS level III, the BMI z-score showed a small median decrease (-0.15) from pre- to five years postoperatively (Table 3).

The median BMI z-score increase between the SDR operation and the follow-up five years later was 0.57 (interquartile range -0.32 to 1.79). The difference between pre- and five-year follow-up BMI z-scores was statistically significant (p < 0.01). The baseline, pre-operative BMI showed a statistically significant effect on the BMI change during five years (p < 0.001), as did age at the start of the follow-up (p < 0.05). GMFCS level and gender did not exhibit a significant effect on the BMI z-score change during the five-year follow-up.

### Under- and overweight

Five of six children with BMI z-score < -2.0 before SDR operation were within typical limits (± 2.0 SD) five years later, while one of the children with typical BMI preoperatively had acquired severe thinness (BMI < -3.0 SD). The two children with preoperative BMI > 2.0 SD were still obese five years later. Six children had acquired obesity defined as BMI z-score > 2.0. Of the eight children with obesity after five years, three had BMI > 2.5 SD and preoperative BMI z-scores of -0.05, 1.90 and 2.61.

Of the three children without complete preoperative measurements, one had BMI z-score > 2.00 at the five-year follow-up and one was severely underweight and stunted at the last contact 18 months postoperatively (Table 2).

In total the number of children with BMI < -2 SD (thinness) decreased and the number with BMI z-score > + 1.0 (overweight) was higher after the five-year follow-up, but these changes did not reach statistical significance. Obesity, defined as BMI < 2.0 SD increased (p < 0.05).

### Age-specific weight, height and BMI development

**Figure 1:**
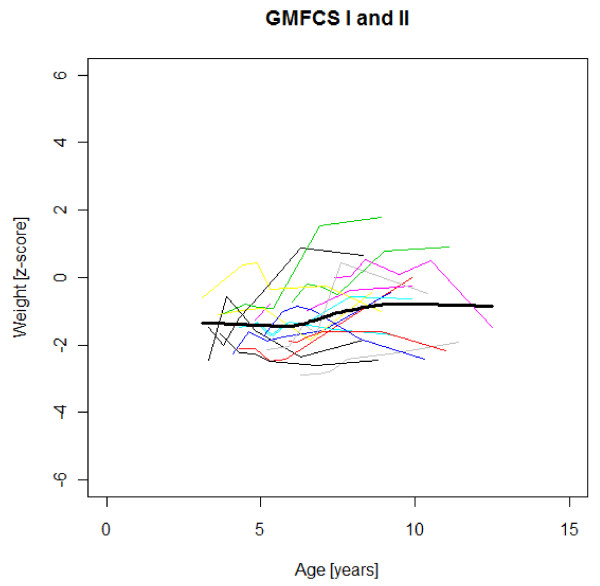
**Weight z-scores in GMFCS I-II**. Weight z-score at different age in 17 children with CP spastic diplegia, GMFCS I-II. Their mean z-score development with age is shown in black.

**Figure 2:**
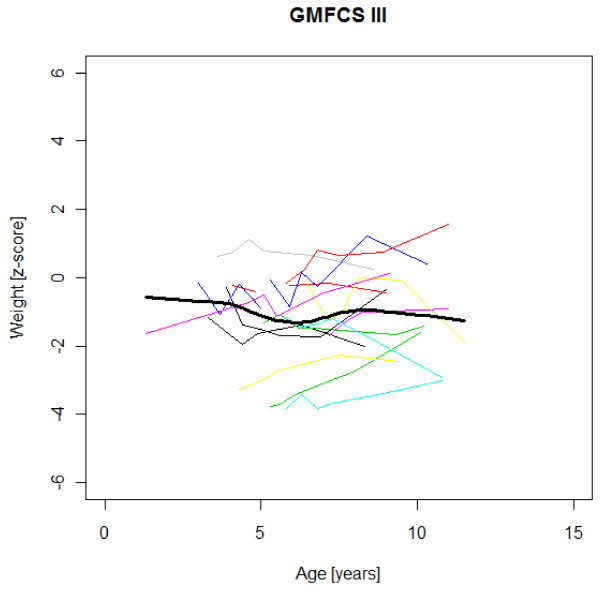
**Weight z-scores in GMFCS III**. Weight z-score at different age in 15 children with CP spastic diplegia, GMFCS III. Their mean z-score development with age is shown in black.

**Figure 3:**
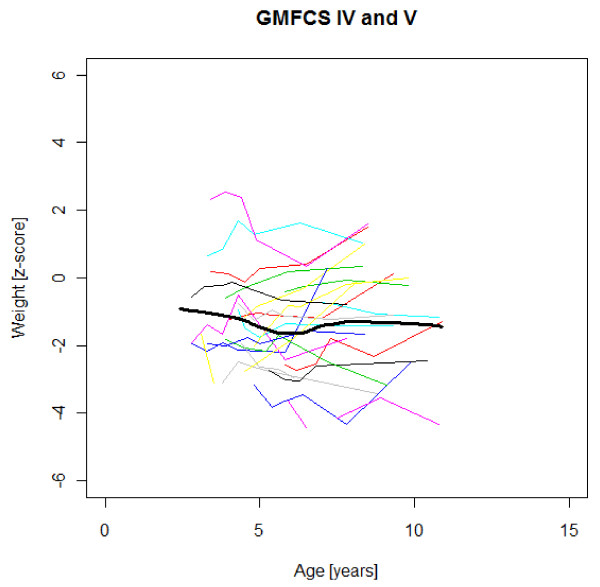
**Weight z-scores in GMFCS IV-V**. Weight z-score at different age in 24 children with CP spastic diplegia, GMFCS IV-V. Their mean z-score development with age is shown in black.

**Figure 4:**
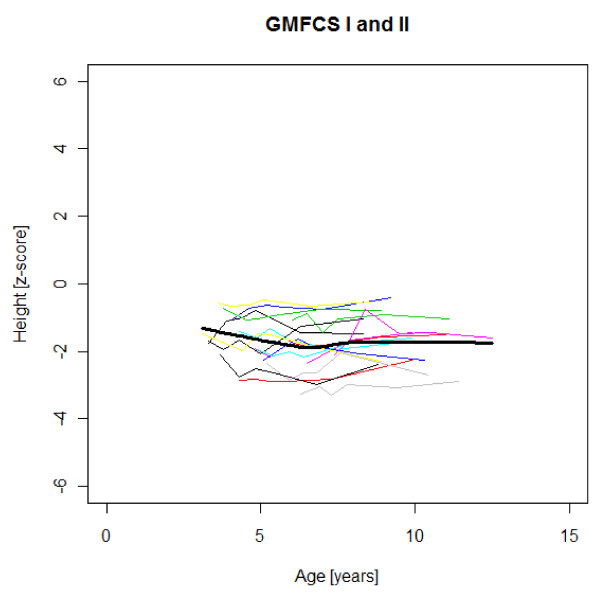
**Height z-scores in GMFCS I-II**. Height z-score at different age in 17 children with CP spastic diplegia, GMFCS I-II. Their mean z-score development with age is shown in black.

**Figure 5:**
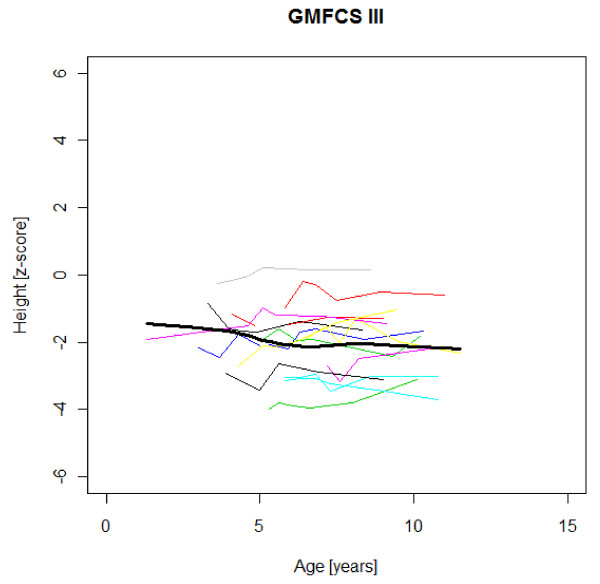
**Height z-scores in GMFCS III**. Height z-score at different age in 15 children with CP spastic diplegia, GMFCS III. Their mean z-score development with age is shown in black.

**Figure 6:**
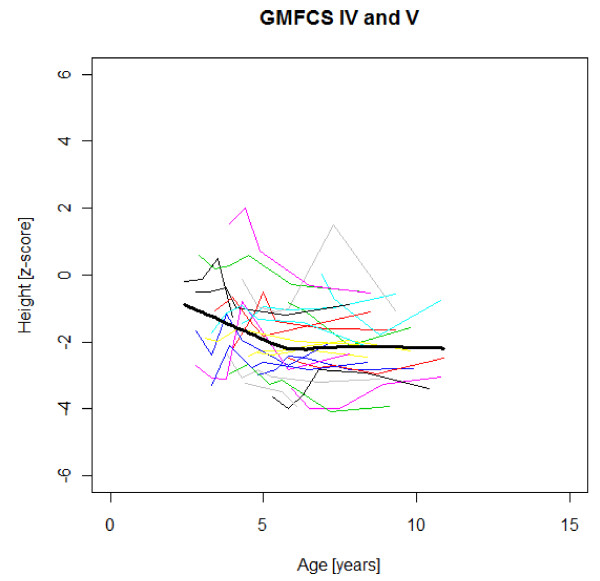
**Height z-scores in GMFCS IV-V**. Height z-score at different age in 24 children with CP spastic diplegia, GMFCS IV-V. Their mean z-score development with age is shown in black.

**Figure 7:**
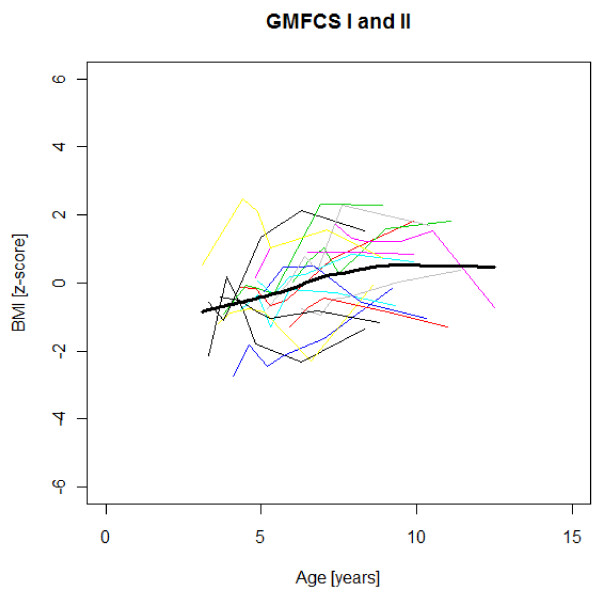
**BMI z-scores in GMFCS I-II**. BMI z-score at different age in 17 children with CP spastic diplegia, GMFCS I-II. Their mean z-score development with age is shown in black.

**Figure 8:**
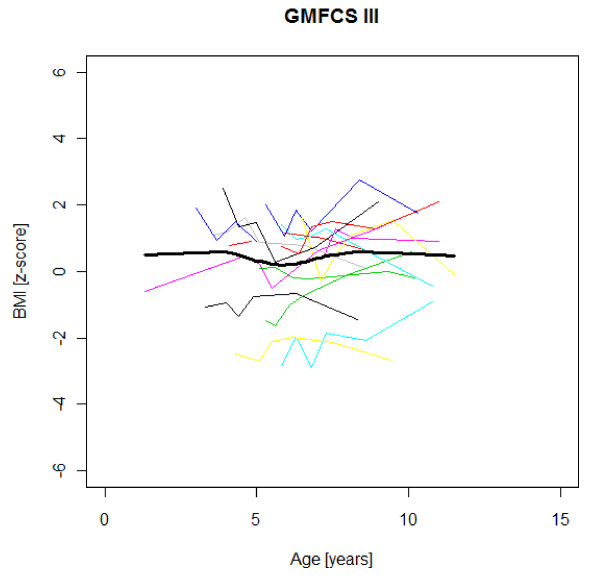
**BMI z-scores in GMFCS III**. BMI z-score at different age in 15 children with CP spastic diplegia, GMFCS III. Their mean z-score development with age is shown in black.

**Figure 9:**
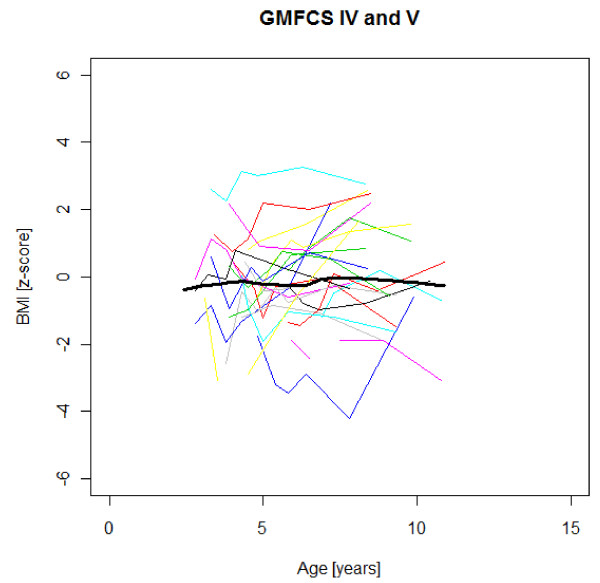
**BMI z-scores in GMFCS IV-V**. BMI z-score at different age in 24 children with CP spastic diplegia, GMFCS IV-V. Their mean z-score development with age is shown in black.

Figures 1,2,3,4,5,6,7,8 &9 show mean weight, height and BMI z-scores for all 56 children with CP diplegia in the different gross motor functional levels at different age during the follow-up, together with individual plots. The children were between 7.4 and 12.4 years of age at the latest measurement. Each child is represented with the same colour in the weight, height and BMI z-scores development lines.

There was an acceleration of mean weight z-scores at six-seven years of age at all GMFCS levels according to Figures 1,2 &3. Height z-score was the most deviant of the three indices at all GMFCS levels. A steep fall in height z-score with age was seen in GMFCS levels IV-V, with more young children at start, in addition to the lowest gross motor function level (Figure 6). The BMI z-score increased at GMFCS levels I-II from the earliest age until ten years, when it levels off including measures from a few children (Figure 7). At the other gross motor function levels the mean BMI z-score was more stable, located above zero in GMFCS III (Figure 8) and below zero at GMFCS IV-V (Figure 9).

## Discussion

### Design and reliability

This is, as far as we know, the first reported series of children with CP of a specified subtype, longitudinally followed with anthropometrics. The study was part of the prospective follow-up during five years of a consecutive, complete series of children with CP spastic diplegia with gross motor function classified at all levels (GMFCS I-V), who were operated with SDR during the first ten years at Lund University Hospital. When introducing SDR in 1993 we chose this practice-based evidence approach to evaluate the outcome [27,28]. The aim was to collect feed-back data to improve our ability to choose the right intervention for each child at the right time [29].

This series may be of value for comparison with other series in the future, even though some of the children were undernourished and/or had other health problems. The presented pre-operative growth data are representative of the CP spastic diplegia subtype at the different GMFCS levels in a developed society and a health care setting with at least fairly good nutritional support, including gastrostomy and available specialists in paediatric gastrointestinal disorders and dieticians. Although the spasticity reduction through SDR may have influenced growth, the postoperative measurements indicate existing growth potential in CP spastic diplegia.

The anthropometric data included in our follow-up were the clinically used standard measurements of height/recumbent length and weight, the most precise anthropometric measures in typically developing children [30]. The reliability of weight measurements is high, also in children with CP. In contrast, direct height/length measurements were regarded as impossible to use in the North American Growth in Cerebral Palsy Project (AGCPP). Knee height was chosen as a proxy at functional levels GMFCS III-V [3]. The children in our study were without any significant scoliosis or severe joint contractures [17]. The measuring was performed by two PTs used to children with CP. The children were well acquainted with the PTs and felt secure in the measuring and training sessions. The PTs could keep the children fully stretched, still and control that ankle, knee, hips and back were in the right position in addition to right position of the heel and head. Therefore we regard the measurements of stature as at least moderately reliable; at the best functional levels GMFCS I-II highly reliable, similar to the reference population.

As GMFCS and subtype-specific reference growth data were unavailable, we chose the growth charts of typically developing healthy children of the same age and gender in our country as a reference, when describing the growth of the study group. The reference population was > 3500 children born at term in 1973-75, i.e. before the "obesity epidemic". They were followed by longitudinal measurements from birth to late adolescence (> 14 times per child) [21,22].

The computer-generated growth chart provided automatic calculations of child age, z-score for every measure and plotting without the common plotting errors of manually completed growth charts [23]. The growth curves were examined by a paediatrician (LW) for each child to identify any errors in the measurements.

### Gender

Twice as many boys as girls were selected for SDR, mainly reflecting gender distribution of CP spastic diplegia in our area (prevalence 1.3/1000 boys and 0.7/1000 girls) [31]. Gender did not exhibit a significant effect on the BMI change during the follow-up (p = 0.75).

### Consequences of weight deviations in CP

Childhood obesity is an increasing problem all over the world. It is associated with a higher chance of premature death and disability in adulthood mainly due to cardiovascular disease, diabetes, cancer and osteoarthritis [32]. Increased obesity prevalence is reported in ambulatory children with CP: from 7.7% in 1994-1997 to 16.5% in 2002-2004 [33], similar to that seen in the general paediatric population [34].

Besides the above-mentioned association with severe long-term health consequences, overweight may cause activity limitations in CP. Simulated weight gain through a heavy belt was shown to have an immediate negative impact on energy costs of walking in children with CP [35]. On the other hand, the NAGCPP study showed that weight < -1 SD was associated with poor health and low societal participation [3,36].

### Stature

Several previous studies have shown that children with CP are shorter and thinner than healthy children with typical development of the same age [e.g. [2,16]]. The relative falling off in stature with age in children with severe functional limitations (GMFCS levels IV-V) in this study is consistent with previous reports [1,3,7,16]. The slight catch-up of median height z-scores during the five years of follow-up in children with standing and walking ability (GMFCS levels I-III) in our study has not been described previously (Table 1). This could be a chance finding. When displayed as mean height z-scores at different ages, no such increase is seen with age (Figures 4-5). The growth potential in childhood CP is unknown, as all studies up to now include children with malnutrition and other health problems.

### BMI

Overweight and obesity are defined as abnormal or excessive fat accumulation that may impair health. BMI provides the most useful population-level measure of overweight and obesity. At the individual level, however, BMI should be considered only as a rough guide, because it may not correspond to the same degree of fatness in different individuals [32]. In children, the proportions of muscle mass and fat mass may vary with age, and it is conceivable that especially great variations may be seen in children with CP, by age and functional level.

### Overweight, obesity and SDR

Children in this series were SDR-operated early in life. Their median weight and BMI z-scores increased with age, and the prevalence of obesity increased from 4% at median age 4.3 years to 15% five years later. If the spasticity reduction after SDR promoted weight gain through less energy requirement, it could be counteracted by increased physical activity, hypothetically the case in the GMFCS III children in this study, who reduced their median BMI. According to Figures 2 and 8 the mean weight and BMI z-scores increased again at six to seven years of age in GMFCS III. At this age all children in Sweden start school.

The accelerating mean weight z-scores at six-seven years of age shown in this study for all GMFCS levels are probably due to more physical inactivity in the school setting than during the pre-school years, with much more focus on activities in locomotion and mobility.

It is questionable whether the increased prevalence of obesity should be viewed as a complication of the SDR operation, as we do not know the "natural course" in non SDR-operated children with spastic diplegia. Available data are scarce, and include other CP subtypes. BMI > +2 SD was present in 6/106 children in Swedish children with bilateral spastic CP (spastic tetraplegia and diplegia) at mean age 7.1 years [16]. The prevalence of obesity, defined as BMI over the 95th percentile, in ambulatory children (mainly spastic hemiplegia and diplegia) with CP was 14-16.5% according to anthropometric measures in a US gait laboratory during 1998-2004 [33]. It is conceivable that the prevalence of obesity accelerates after 5-6 years of age in children with CP as in other children [34].

## Conclusions

The presented growth data for children with CP spastic diplegia with different GMFCS levels are the first available subtype-specific data of value for comparison with other series. As some of the children were undernourished and/or had other health problems, the growth potential is probably higher in optimal circumstances.

The children with spastic diplegia were all shorter than the reference population. The median height z-score was -1.92, and was unchanged after five years for the whole group. There was a tendency towards an increasing stunting with age in severe gross motor dysfunction (GMFCS levels IV-V) and the opposite, a slight catch-up of median height in children with walking ability (GMFCS levels I-III).

A significant increase in BMI z-scores was found during five years of follow-up. For many children the weight and BMI increase was highly desirable. The proportion of children with underweight, thinness, decreased (n.s.), while the prevalence of obesity increased (p < 0.05). The question about increased weight gain velocity as a side effect of SDR is unanswered at present. It may be that the entire weight/BMI z-score increase with age was part of the general "obesity epidemic".

Severe weight deviations in CP are common and should be prevented to improve health, mobility, activity and participation. Regular monitoring of weight and height or proxy measures, entered into growth charts, and timely interventions to promote healthy growth, are necessary.

These are the first available subtype- and GMFCS-specific growth data. The growth potential according to these data should be regarded as a minimum. Until reliable GMFCS- and subtype-specific growth-charts from adequately nourished and otherwise healthy children with CP are available, we recommend the use of growth data for typically developing children as a reference.

## Competing interests

The authors have no financial interest in this study, which was performed as part of a clinical follow-up programme after the introduction of SDR as a treatment option in Lund 1993.

## Authors' contributions

EN, ALJ and LW planned, administered assessments/measurements, and analysed the results together with PW, who chose, described and performed the statistics. LW wrote and revised the manuscript with the active assistance of all authors.

## Pre-publication history

The pre-publication history for this paper can be accessed here:

http://www.biomedcentral.com/1471-2377/10/57/prepub
